# Fresh-frozen Complete Extensor Mechanism Allograft versus Autograft Reconstruction in Rabbits

**DOI:** 10.1038/srep22106

**Published:** 2016-02-25

**Authors:** Guanyin Chen, Hongtao Zhang, Qiong Ma, Jian Zhao, Yinglong Zhang, Qingyu Fan, Baoan Ma

**Affiliations:** 1 Department of Orthopedics, Tangdu Hospital, Fourth Military Medical University, 569 Xinsi Road, Xi’an 710038, China

## Abstract

Different clinical results have been reported in the repair of extensor mechanism disruption using fresh-frozen complete extensor mechanism (CEM) allograft, creating a need for a better understanding of fresh-frozen CME allograft reconstruction. Here, we perform histological and biomechanical analyses of fresh-frozen CEM allograft or autograft reconstruction in an *in vivo* rabbit model. Our histological results show complete incorporation of the quadriceps tendon into the host tissues, patellar survival and total integration of the allograft tibia, with relatively fewer osteocytes, into the host tibia. Vascularity and cellularity are reduced and delayed in the allograft but exhibit similar distributions to those in the autograft. The infrapatellar fat pad provides the main blood supply, and the lowest cellularity is observed in the patellar tendon close to the tibia in both the allograft and autograft. The biomechanical properties of the junction of quadriceps tendon and host tissues and those of the allograft patellar tendon are completely and considerably restored, respectively. Therefore, fresh-frozen CEM allograft reconstruction is viable, but the distal patellar tendon and the tibial block may be the weak links of the reconstruction. These findings provide new insight into the use of allograft in repairing disruption of the extensor mechanism.

Extensor mechanism disruption, either from severe fracture or tendon rupture, typically occurs after or during total knee arthroplasty (TKA), with an incidence of less than 2.5%^1^,^2^,^3^,^4^,^5^,^6^. Although the complication is infrequent, its treatment is technically complex and mainly depends on the etiology and anatomical location of the injury and the viability of the local host structures. Direct primary repair provides encouraging results only in native knees with an acute and fresh injury^7^,^8^,^9^,^10^. Local autogenous tissues have been reported to augment direct primary repair^3^,^11^,^12^,^13^, but the availability and quality of these tissues are usually unsatisfactory due to previous knee surgeries. The use of allograft tissues has shown promise in the reconstruction of extensor mechanism failure. Bone-patellar tendon-bone allograft^14^,^15^,^16^,^17^ and Achilles tendon bone block allograft^6^,^18^,^19^,^20^,^21^,^22^,^23^ have provided satisfactory results for patients with an adequate patella and patellar component. Fresh-frozen complete extensor mechanism (CEM) allograft consisting of the quadriceps tendon, patella, patellar tendon and anterior tibial tubercle is suitable for patients with insufficient or no patella, or other complicated lesions^4^,^6^,^24^,^25^,^26^,^27^,^28^,^29^,^30^,^31^.

Fresh-frozen CEM allograft reconstruction was first demonstrated in patellar tendon rupture after TKA^24^. Despite the early promising results, extensor lag subsequently occurred in some patients^25^. The technique was modified by tightly tensioning the allograft in full extension^28^, and the clinical results improved^4^,^6^,^29^,^30^. However, high failure rate of reconstruction using fresh-frozen CEM allograft has also been reported^27^,^31^. Four of seven reconstructions were revised postoperatively, and comparisons of the knee score and the extensor lag with the preoperative values showed no significant differences^27^. Moreover, proximal tibial shaft fracture resulting from stress riser has also been reported, especially in long-term follow-up^31^. Allograft bone block and Achilles tendon have been shown to be incorporated into host tissues^14^,^15^,^16^,^17^,^19^,^24^. However, no published studies have systematically presented histological and biomechanical evidences for the viability of fresh-frozen CEM allograft used for extensor mechanism reconstruction.

This study was designed to establish an *in vivo* rabbit model of fresh-frozen CEM allograft or autograft reconstruction and to investigate whether the allograft is a viable alternative for extensor mechanism reconstruction by conducting histological and biomechanical analyses during the first six months after surgery. A rabbit model of fresh-frozen CEM allograft or autograft reconstruction was first established. Quantitative analysis of cellularity and qualitative evaluation of vascularity were performed using Indian-ink perfusion and hematoxylin and eosin (H&E) staining. The strength of the junction of the quadriceps tendon and the soft tissues and the strength of the patellar tendon were measured using biomechanical test. We hypothesized that fresh-frozen CEM allograft reconstruction may be viable because the allograft would be incorporated into the host tissues even during a low remodeling process. Our study provides evidences for continuity between the surviving allograft and the surrounding tissues, despite a reduced number of osteocytes in the allograft tibia, the main blood supply from the infrapatellar fat pad, and significant restoration of the biomechanical properties of the allograft tendons, despite their low cellularity. These results indicate that fresh-frozen CEM allograft is a viable alternative for extensor mechanism reconstruction, but the distal patellar tendon and the tibial block may be the weak links of the reconstruction.

## Results

The 84 rabbits included in this study all survived the experiment. One specimen was excluded from the autograft group (n = 28) at 4 weeks postoperatively because of loose suture and infection. One specimen in the allograft group (n = 56) and one specimen in the autograft group were excluded at 12 weeks postoperatively due to improper performance of the biomechanical test.

### Complete incorporation of the quadriceps tendon into the host tissues, patellar survival and total integration of the allograft tibia, with relatively fewer osteocytes, into the host tibia

**Figure 1:**
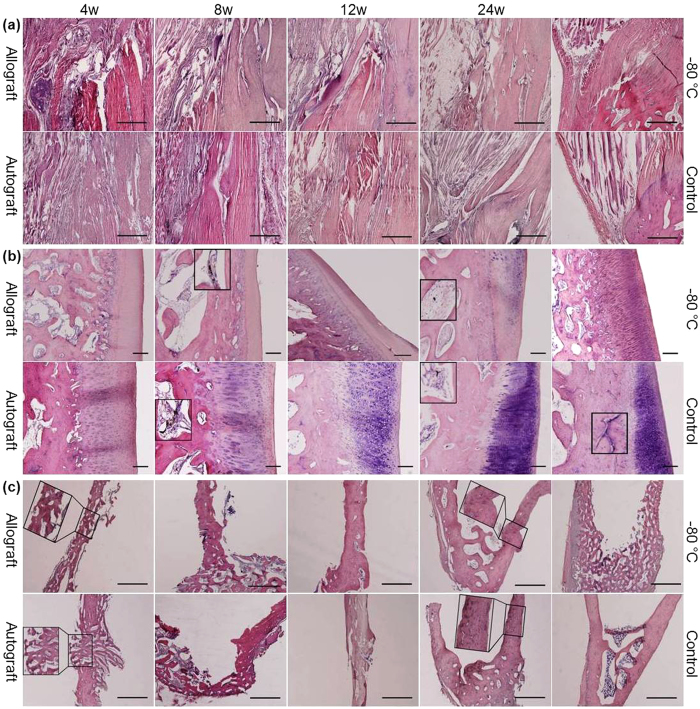
Photomicrographs of the junction of the quadriceps tendon and the soft tissues, patella and tibial insertion site. H&E staining of the junction of the quadriceps tendon and the soft tissues (**a**) (scale bar, 1 mm), the patella (**b**) (scale bar, 200 μm) and the tibial insertion site (**c**) (scale bar, 1 mm) in the allograft and autograft groups at 4, 8, 12 and 24 weeks postoperatively and in the −80 °C and control groups. Inserts show higher magnification of vessels in (**b)** and higher magnification of tibial insertion sites in (**c**).

As shown in Fig. 1a, in the allograft group, there were large empty spaces in the junction of the quadriceps tendon and the host tissues at 4 weeks. Vessels and cells were indistinct in the proximal quadriceps tendon compared with the hypervascularity and hypercellularity observed in the autograft group. At 8 weeks, in the allograft group, vessels extended from the host quadriceps muscles and tendon into the proximal quadriceps tendon, and a large number of cells infiltrated into the proximal quadriceps tendon, By contrast, in the autograft group, the proximal quadriceps tendon was in close contact with the quadriceps muscles. At 12 weeks, the cellularity and vascularity of the proximal quadriceps tendon decreased in both the allograft and autograft groups, but the allograft quadriceps tendon and the autograft quadriceps tendon were partly and totally connected to the surrounding tissues, respectively. At 24 weeks, the quadriceps tendon was completely incorporated into the surrounding tissues in both the allograft and autograft groups. However, the histological properties in the autograft group were similar to those in the control group.

As shown in Fig. 1b, at 4 weeks, a thin layer of chondrocytes, no osteocytes, and numerous small empty spaces between the trabeculae occurred in the allograft patella, whereas in the autograft group, a thick layer of chondrocytes, osteocytes on the periphery of the trabeculae, and small empty spaces between the trabeculae were observed. At 8 weeks, a thinner layer of chondrocytes, large empty spaces and only a few vessels were found in the allograft patella, By contrast, in the autograft group, increased number of chondrocytes and osteocytes, relatively small empty spaces and many vessels were observed. At 12 weeks, inflammatory cells were seen in a thin layer of chondrocytes in the allograft patella, whereas in the autograft group, additional increases in the number of chondrocytes and osteocytes were found. At 24 weeks, no osteocytes and very few thin vessels were observed in the allograft patella. However, the number of chondrocytes in the allograft group increased but remained lower than that in the autograft and control groups. Thin vessels were still observed in the autograft group, which had histological properties similar to those in the control group, except for a reduced number of osteocytes.

As shown in Fig. 1c, at the tibial block insertion site, newly woven bony incorporation and vessels appeared at 4 weeks, increased bone ingrowth was observed at 8 and 12 weeks, and compact bone integration characterized by Indian-ink perfusion of Haversian canals was found at 24 weeks in both the allograft and autograft groups. However, at 24 weeks, tibia in the allograft group had relatively fewer osteocytes than tibia in the autograft group, which had histological properties similar to those in the control group.

### Decreased and delayed cellularity in the allograft group but with a similar cellular distribution to that in the autograft group

**Figure 2:**
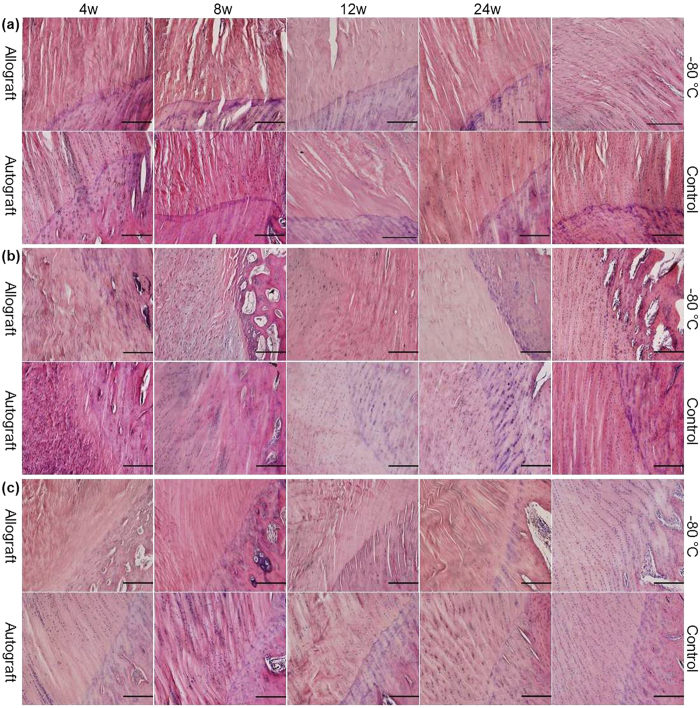
Photomicrographs of the quadriceps tendon close to the patella, the patellar tendon close to the patella and the patellar tendon close to the tibia. H&E staining of the central portions of the quadriceps tendon close to the patella (**a**), the central portions of the upper part of the patellar tendon close to the patella (**b**) and the central portions of the lower part of the patellar tendon close to the tibia (**c**) in the allograft and autograft groups at 4, 8, 12 and 24 weeks postoperatively and in the −80 °C and control groups. The scale bar represents 200 μm in (**a–c**).

**Figure 3:**
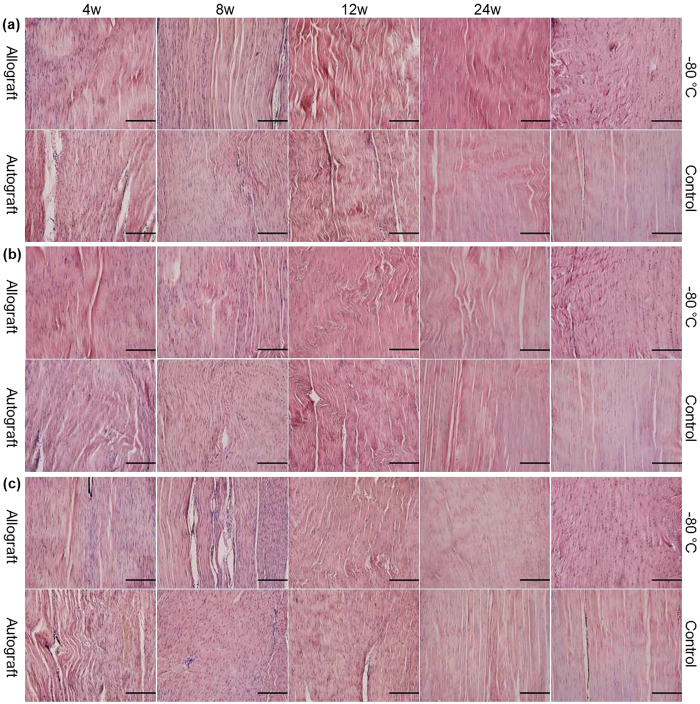
Photomicrographs of the dorsal, central and ventral patellar tendons. H&E staining of the dorsal (**a**), the central (**b**) and the ventral (**c**) portions of the distal of the upper part of the patellar tendon in the allograft and autograft groups at 4, 8, 12 and 24 weeks postoperatively and in the −80 °C and control groups. The scale bar represents 200 μm in (**a**–**c**).

At 4 weeks, the central portion of the quadriceps tendon close to the patella (Fig. 2a) and the central portion of the patellar tendon close to the tibia (Fig. 2c) were almost acellular in the allograft group, whereas cells could be observed in the autograft group. In the allograft group, cells were visible in the central portion of the patellar tendon close to the patella (Fig. 2b) and in the dorsal (Fig. 3a) and ventral patellar tendons (Fig. 3c), but there were relatively fewer cells in these tissues than in the autograft group. At 8 weeks, the cellularity of the central portion of the quadriceps tendon close to the patella and the cellularity of the entire patellar tendon, excluding the patellar tendon close to the tibia, substantially increased in the allograft group, whereas hypercellularity remained present in the autograft group. At 12 weeks, in both the allograft and autograft groups, the cellularity decreased, but the cellular distribution was more homogeneous and collagen realignment improved. At 24 weeks, in both the allograft and autograft groups, a continuous decrease in cellularity was observed, but the cells were longitudinally reorganized in regularly aligned collagen fibers. The pattern in the autograft group more closely resembled that in the control group.

As shown in Table 1, the highest cellularity was observed at 8 and 4 weeks in the allograft and autograft groups, respectively, in almost all parts. The cellularity in almost all parts in the allograft group was significantly lower than that in the autograft group. The cellularity in almost all parts in both the allograft and autograft groups was significantly lower than that in the control group at 24 weeks. The cellularity in all parts in the −80 °C group was significantly lower than that in the control group. In the vertical axis, compared with the cellularity of the patellar tendon close to the patella, the cellularity of the patellar tendon close to the tibia was significantly lower at all time points in both the allograft and autograft groups, but the cellularity of the central patellar tendon showed no significant differences at all time points in the allograft group and at almost all time points in the autograft group. However, in the control group, compared with the cellularity of the patellar tendon close to the patella, the cellularity of the patellar tendon close to the tibia was not significantly different, but the cellularity of the central patellar tendon was significantly lower. In the sagittal axis, compared with the cellularity of the dorsal patellar tendon, the cellularity of the ventral patellar tendon was significantly higher at all time points in both the allograft and autograft groups, but the cellularity of the central patellar tendon was significantly lower at 4, 8 and 12 weeks in the allograft group and at 4 weeks in the autograft group. However, no significant difference in cellularity was observed between the dorsal, ventral and central patellar tendons in the control group.

### Robust but delayed revascularization in the allograft patellar tendon

**Figure 4:**
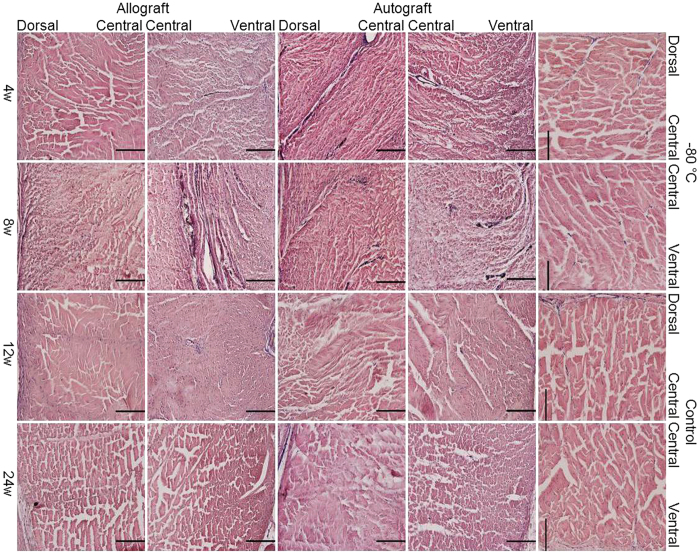
Robust but delayed revascularization in allograft patellar tendon. H&E staining of the dorsal, central and ventral portions of the middle part of the patellar tendon (transverse section) after India-ink perfusion in the allograft and autograft groups at 4, 8, 12 and 24 weeks postoperatively and in the −80 °C and control groups. The scale bar represents 200 μm.

As shown in Fig. 4, in the middle part of the patellar tendon, there were no visible vessels in the dorsal and central portions and only a few thin vessels in the ventral portion in the allograft group at 4 weeks. However, bold vessels extended into the central portion from the dorsal and ventral portions in the autograft group. At 8 weeks, many bold vessels, especially those from the ventral portion extended into the central portion in the allograft group, whereas the vessels were visible in the dorsal, central and ventral portions in the autograft group. At 12 weeks, very few vessels were observed in the dorsal and central portions, and those identified in the ventral portion were thin and short in both the allograft and autograft groups. At 24 weeks, almost no vessels were observed in the dorsal and central portions, but there were still short vessels in the ventral portion in both the allograft and autograft groups.

### Complete restoration of the biomechanical properties of the junction of quadriceps tendon and host tissues and considerable restoration of those of the allograft patellar tendon

As shown in Table 2, in the junction of the quadriceps tendon and the soft tissues, all parameters but the cross-sectional area of the quadriceps tendon were significantly lower at 4 weeks than those at 8, 12 and 24 weeks in both the allograft and autograft groups. Compared with those in control group, all parameters but the cross-sectional area of the quadriceps tendon in both the allograft and autograft groups were significantly lower at 4 weeks, but no significant differences were observed at 24 weeks. Compared with those in the autograft group, the maximum load to failure, stiffness at failure and maximum stress were all significantly lower, but the cross-sectional area of the quadriceps tendon was significantly larger in the allograft group at 8 weeks. The cross-sectional area of the quadriceps tendon at 24 weeks was significantly smaller than that at 4 weeks in the allograft group, and it was significantly smaller in both the allograft and autograft groups than in the control group.

In the patellar tendon, almost all parameters were significantly lower at 4 weeks than those at 8, 12 and 24 weeks in both the allograft and autograft groups. Compared with those in the autograft group, almost all parameters in the allograft group were significantly lower at all time points. Compared with those in the control group, almost all parameters in both the allograft and autograft groups were significantly lower at all time points. In both the allograft and autograft groups, the cross-sectional area of the patellar tendon was significantly smaller at 24 weeks than at 4 weeks, but it was significantly larger than that in the control group at all time points. The maximum load to failure, stiffness at failure, maximum stress and elastic modulus in the allograft group at 24 weeks exhibited 73.6%, 69.5%, 65.8% and 63.9%, respectively, of those in the autograft group and 70.7%, 61.9%, 54% and 43% , respectively, of those in the control group. The maximum load to failure, stiffness at failure, maximum stress and elastic modulus in the autograft group at 24 weeks exhibited 89%, 82.9%, 77.1% and 62.1%, respectively, of those in the control group.

## Discussion

Although different findings have been reported in the repair of extensor mechanism disruption using fresh-frozen CEM allograft, few, if any, studies have conducted histological and biomechanical analyses of the remodeling of the allograft. In this study, we chose rabbits to perform fresh-frozen CEM allograft or autograft reconstruction because remodeling of cortical bone in rabbits has been shown to be similar to that in humans^32^, although rabbits do not have an extended resting position like elephants^33^. We show that both fresh-frozen CEM allograft and autograft reconstructions produce satisfactory results. Decreased cellularity after freezing and no immunological rejection in fresh-frozen CEM allograft reconstruction are consistent with previous findings demonstrating that freezing can perish tendon cells^34^ and reduce the antigenicity^35^ and immunoreactivity^36^ of the allograft. Complete incorporation of the allograft quadriceps tendon into the host tissues is similar to a previous finding showing that the junction of the Achilles tendon and the host muscles was in continuity^19^. The rabbits receiving fresh-frozen CEM allograft (Supplementary Videos S1–S4), like the rabbits receiving CEM autograft (Supplementary Videos S5–S8), are able to move freely and maintain their daily activities at each postoperative time point.

We show that the patterns of vascular and cellular distributions in the allograft patellar tendon in the sagittal and vertical axes are similar to those in the autograft patellar tendon, although they differ from those in the control. In the sagittal axis, the main blood supply for the patellar tendon in both the allograft and autograft groups is from the infrapatellar fat pad. This is in agreement with previous studies demonstrating that the vessels of the patellar tendon used for anterior cruciate ligament reconstruction were typically obtained from the infrapatellar fat pad surrounding the graft^36^,^37^,^38^. Three remodeling stages (degeneration, proliferation, maturation) have been reported in the use of the patellar tendon for anterior cruciate ligament reconstruction^36^,^37^,^38^,^39^,^40^,^41^,^42^,^43^. But our study further reveals that the highest vascularity and cellularity are observed in the ventral portion of the patellar tendon, followed by the dorsal portion and the central portion. The relatively lower vascularity and cellularity in the dorsal portion of the patellar tendon in the allograft group may be the results of the removal of synovial membrane before allograft implantation. In the vertical axis, the highest cellularity is observed in the patellar tendon close to the patella, whereas the lowest cellularity is present in the patellar tendon close to the tibia in both the allograft and autograft groups. The consistently low cellularity in the distal patellar tendon may be explained by the relatively reduced amount of tissues covering the implantation and a greater distance from infrapatellar fat pad, which may contribute to one weak link of the reconstructions. This is probably a histological foundation of patellar tendon disruption occurred after fresh-frozen CEM allograft reconstruction in clinical practices^27^. Our results also show that the vascularity and cellularity in the allograft group is reduced, and their development is delayed, compared with those in the autograft group. A possible explanation is the immediate *in situ* implantation of the autograft, resulting in the absence of a degeneration stage in the parts close to the infrapatellar fat pad.

Although the appearance of abrasion and osteoporosis (*i.e.*, thin layer of chondrocytes and large spaces between the trabeculae) in the allograft patella, revascularization and the increased number of chondrocytes indicate that the patella is survived. The complete integration of the allograft tibia into the host tibia is similar to the findings of some previous studies which found the healing of the allograft bone block to the host tissue at all tibial junctions^14^,^15^,^16^,^17^,^19^,^24^. However, the reduced number of osteocytes in the allograft tibia may result in the other weak link of freshen-frozen CEM allograft reconstruction, which is probably another cause of fracture at the distal of the allograft tibial block, apart from the reported stress riser resulting from the same level of the allograft bone block and the distal of the tibial component of prosthesis^31^.

The complete restoration of biomechanical properties of the junction of the quadriceps tendon and the soft tissue in both the allograft and autograft groups is better than that shown in some previous reports demonstrating only partial restoration of the biomechanical properties of tendons inserted into bones^36^,^43^,^44^,^45^,^46^, which may indicate that tendons can be incorporated more easily into soft tissues than into bones. The relatively limited biomechanical properties of the allograft patellar tendon may be due to the histological evidence for low cellularity, especially in the distal patellar tendon close to the tibia. However, the time-dependent increase of biomechanical results suggests that the biomechanical properties of the allograft patellar tendon could further improve.

There are several limitations in this study. The first limitation is that normal knee, not knee with TKA, was evaluated. In clinical settings, fresh-frozen CEM allograft is mainly used to repair the extensor mechanism disruption that typically results from TKA. However, our study is a step towards better understanding of fresh-frozen CEM allograft reconstruction in patients with TKA, because rabbits with normal knees were performed the same fresh-frozen CEM allograft reconstruction to patients with TKA and had similar host tissues to patients with TKA to cover the allograft in the present study. The second limitation is that only qualitative evaluation of vascularity by India-ink perfusion was used to determine the main blood supply of the patellar tendon. Further immunohistochemistry for vascular markers, death-life-staining, proteoglycan content staining and collage fiber orientation staining would further elucidate the remodeling process. The third limitation is that only the use of fresh-frozen allograft was assessed. Other types, such as freeze-dried and radiated allografts, may produce different histological and biomechanical results and are worth investigating in future studies.

In summary, we have established for the first time an *in vivo* rabbit model of fresh-frozen CEM allograft or autograft reconstruction and performed histological and biomechanical analyses. The histological results show that the allograft is completely incorporated into the host tissues, with relatively fewer chondrocytes in the patella and relatively fewer osteocytes in the tibial block, and that vascularity and cellularity in the allograft are lower, especially in the distal patellar tendon, and delayed but display similar distributions to those in the autograft. The biomechanical results show that the biomechanical properties of the junction of the allograft quadriceps tendon and the host tissues and those of the allograft patellar tendon are completely and considerably restored, respectively, relative to those of the control. Therefore, fresh-frozen CEM allograft is a viable alternative for the reconstruction of extensor mechanism disruption, but the distal patellar tendon and the tibial block may be the weak links of the reconstruction.

## Methods

### Study design

A total of 84 New Zealand rabbits (2.2–3.5 kg, 4–8 months old) were used in this experiment. Each rabbit was checked for general health, housed in commercial animal cages (49 cm × 35 cm × 32 cm) and had free access to food and water. The experimental protocol was approved by the Animal Care and Use Committee of the Fourth Military Medical University. The methods were carried out in accordance with the approved guidelines. Every effort was made to improve the comfort of the rabbits.

The rabbits were divided into the allograft group (n = 56) and the autograft group (n = 28). In the allograft group, 28 rabbits were used to harvest right CEMs. The 28 right CEMs were soaked in gentamicin solution (80,000 U) and immediately stored in sterile bags at −80 °C (MDF-U73V, SANYO Electric Co., Ltd. Japan) for one week. Each of the remaining 28 rabbits received one right fresh-frozen CEM allograft. In the autograft group, 28 rabbits underwent right CEM autograft reconstruction. At 4, 8, 12 and 24 weeks postoperatively, 2 rabbits were used from each group for Indian-ink perfusion and histological analysis, and 5 rabbits were used for biomechanical test. From each rabbit, both CEMs were harvested, and the left CEM served as the control group. The left CEMs frozen at −80 °C for 7 days served as the −80 °C group. Postoperatively, the rabbits were returned to their own cages, softly touched every day, and given an intramuscular injection of cefazolin sodium (0.25 g) for 7 consecutive days. No analgesics were administered. The cast was removed after 3 weeks of immobilization.

### Allograft and autograft implantation

**Figure 5:**
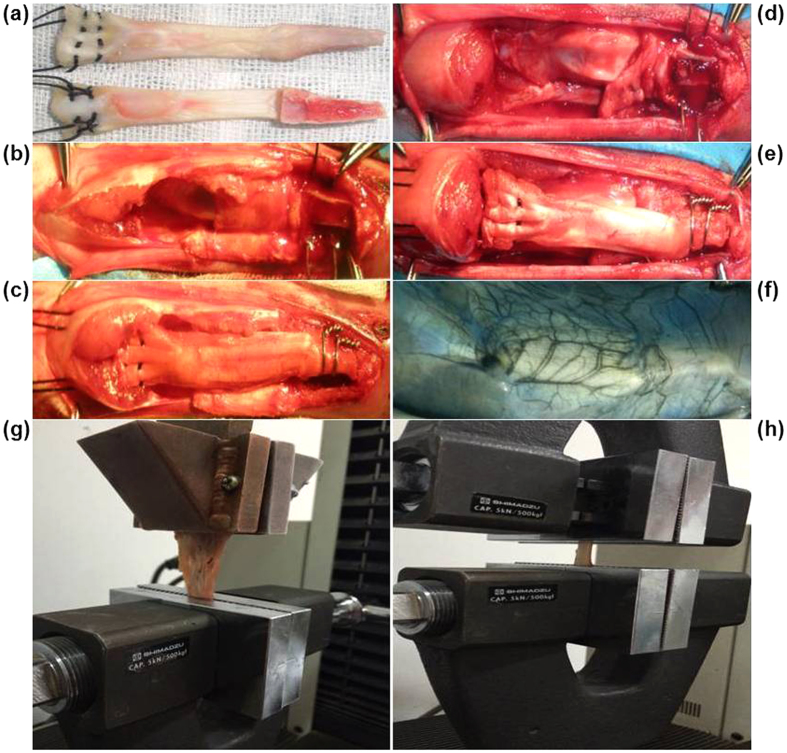
Surgical procedure for CEM implantation, India-ink perfusion of the control and the postoperative biomechanical test. (**a**) Dorsal and ventral views of sutures placed in a modified Krackow fashion in the lateral and medial aspects of the quadriceps tendon. (**b**) Preparation for allograft implantation. (**c**) Allograft implantation with full extension under the host quadriceps muscles and distal fixation. (**d**) Preparation for autograft implantation. (**e**) *In situ* implantation of the autograft with full extension and distal fixation. (**f**) An angiograph of the left CEM after India-ink perfusion. (**g**) Biomechanical test of the junction of the quadriceps tendon and the soft tissues. (**h**) Biomechanical test of the patellar tendon.

The technique used for fresh-frozen CEM allograft reconstruction was based on that of previous studies^28^,^47^. Surgery was performed in a clean dissecting room after ultraviolet radiation. At the allograft preparation stage, the right leg of the rabbit, held in a supine position, was depilated using 8% Na_2_S under anesthesia [40 mg/kg body weight (b. w.), sodium pentobarbital (Merck KGaA, Darmstadt, Germany), i.p.]. A median knee incision was performed and the quadriceps tendon was first severed 1.5 cm above the patella. The soft tissues alongside the patella and the patellar tendon were then separated. Finally, the CEM was harvested following the creation of a tibial bone block that was 1.2 cm long, 0.5 cm wide and 0.4 cm thick. Before implantation, the fresh-frozen CEM allograft stored in a sterile bag was bathed in 37 °C water for 5 minutes, and 2 number-5 nonabsorbable sutures [Johnson & Johnson Medical (China) Ltd.] were placed along the lateral and medial aspects of quadriceps tendon in a modified Krackow fashion^48^ (Fig. 5a). During implantation, the host extensor mechanism was split longitudinally in the midline following the removal of the patella. The proximal of the host tibial trough was 0.3 cm below the anterior tibial component to prevent migration of the graft. The host tibial trough was rectangular (1.2 cm in length, 0.5 cm in width, and 0.4 cm in depth) to provide a close fit to the tibial bone block of the allograft (Fig. 5b). Two stainless steel wires (Φ = 0.5 mm) were placed at the base of the trough and twisted over the bone block. The quadriceps tendon was pulled tightly and proximally under the host quadriceps tendon with the knee in full extension (Fig. 5c). The allograft was then sutured beneath the host quadriceps tendon and distal tissues in a vest-over-pants fashion with the allograft covered as much as possible. The wound was then sutured, and a plaster cast was applied with the knee in full extension. At the autograft implantation stage, the right CEM was cut and immediately placed *in situ* (Fig. 5d,e).

### Indian-ink perfusion

Rabbits were anesthetized (as described above) before depilation of the abdomen and the legs. Subsequently, a median abdominal incision was created, followed by the exposure of the abdominal aorta and the inferior vena cava and the ligation of their branches. Before a syringe needle was inserted into the distal of the abdominal aorta, the proximal of the abdominal aorta and the inferior vena cava were tied off. The syringe needle was secured in the vessel, and the distal of the inferior vena cava was transected. Approximately 500 mL of heparinized saline (25000 IU/L) was needed to perfuse the abdominal aorta at a rate of 4 ml/min until the blood outflow from the distal of the inferior vena cava stopped and the fluid became clear. Next, a 10% formalin solution and a 10% Indian-ink solution (Beijing Solarbio Science & Technology Co. Ltd., China) filtered through quantitative filter paper (slow speed, Φ = 1–3 μm) were serially perfused for 30 minutes each at the same rate as the saline perfusion. At the end of the India-ink perfusion, the distal of the inferior vena cava was tied off. An angiograph of the left CEM is shown in Fig. 5f.

### Histological analysis

The Indian-ink perfused CEM, including the proximal and distal of the adjacent tissues, was first fixed in Bouin’s fluid for 24 hours. Subsequently, it was divided into 5 parts after being transected along the midline of the patella and tibia block: 1) the junction of the quadriceps tendon and the soft tissues, the quadriceps tendon and half of the patella; 2) the upper part of the patellar tendon and half of the patella; 3) the middle part of the patellar tendon; 4) the lower part of the patellar tendon and half of the tibial block; 5) the tibial block insertion site. The specimens, excluding the middle part of the patellar tendon, were decalcified in 10% ethylenediaminetetraacetic acid for 4 weeks. Next, they were dehydrated in a graded ethanol solutions and embedded in paraffin. Five sections with a thickness of 5 μm, spaced at an interval of 50 μm, were obtained from each specimen (LEICA RM2235, Germany), stained with H&E and evaluated by 2 of the authors (G.C. and H.Z.) in a blinded manner.

All of the sections were observed at different magnifications for the quantitative and qualitative analyses of cellularity and vascularity using a high-resolution microscope (Olympus BX40, Japan). The cellularity of the quadriceps tendon and the patellar tendon were evaluated in longitudinal and median section. The vascularity of the middle part of the patellar tendon was assessed in transverse section. Each section was divided into a ventral portion, a central portion and a dorsal portion. In the central portions of the upper part of the patellar tendon close to the patella and the lower part of the patellar tendon close to the tibia, one region of interest (0.5651 mm^2^) in each section was identified and the number of cells was counted. At the distal of the upper part (or the proximal of the lower part) of the patellar tendon, one region of interest (0.5651 mm^2^) from the dorsal, central and ventral portions of each section was analyzed, and the cellularity of the dorsal, central and ventral patellar tendons was calculated. In addition, the cellularity of the central portion of the quadriceps tendon close to the patella was calculated. Histological remodeling processes of the junction of the quadriceps tendon and the soft tissues, the patella and the tibial block insertion site were described.

### Biomechanical analysis

After harvesting CEM, including the proximal and distal of the adjacent tissues, the sutures inside the graft were cut off, and the 2 stainless steel wires were carefully removed. The specimens were then wrapped in saline-soaked gauze and stored at −20 °C. Before the biomechanical test, they were kept at 4 °C overnight. Each specimen was stretched twice to evaluate the strength of the junction of the quadriceps tendon and the soft tissues, as well as the strength of the patellar tendon. After measuring the cross-sectional area of the middle part of both the quadriceps tendon and the patellar tendon, the strength of the junction of the quadriceps tendon and the soft tissues was tested. The soft tissues were mounted on a custom-made copper clamp and secured by deep freezing with liquid nitrogen (Fig. 5g). The patella was tightly mounted on an aluminum clamp of a biomechanical testing machine (SPL-10 KN, Shimadzu, Japan). The test was applied along the vertical axis, and the specimen was loaded until failure at a displacement rate of 10 mm/min. The strength of the patellar tendon was then tested with the patella and the tibial bone block mounted on aluminum clamps (Fig. 5h). The maximum load to failure, cross-sectional area, stiffness at failure, maximum stress and elastic modulus were calculated.

### Statistical analysis

SPSS version 16.0 was used in this study. Data were considered to be normal distribution and homogenous variances when *P* > 0.1 using the Shapiro-Wilk test and Levene’s test. All data are presented as the mean ± standard error. Data with normal distribution and homogenous variances were compared using the Student’s *t*-test. Otherwise, the Mann-Whitney *U*-test was employed. *P* < 0.05 was considered significant.

## Additional Information

**How to cite this article**: Chen, G. *et al.* Fresh-frozen Complete Extensor Mechanism Allograft versus Autograft Reconstruction in Rabbits. *Sci. Rep.* **6**, 22106; doi: 10.1038/srep22106 (2016).

## Supplementary Material

Supplementary Information

Supplementary Video S1

Supplementary Video S2

Supplementary Video S3

Supplementary Video S4

Supplementary Video S5

Supplementary Video S6

Supplementary Video S7

Supplementary Video S8

## Figures and Tables

**Table 1:** Comparison of the cellularity (per mm^2^) of the allograft, autograft, −80 °C and control groups (mean ± standard error).

	Quadriceps tendon close to the patella	Patellar tendon close to the patella	Central patellar tendon	Patellar tendon close to the tibia	Dorsal patellar tendon	Ventral patellar tendon
Allo 4W	130 ± 9^☆Δ^	499 ± 56^☆Δ^	469 ± 35^☆Δ‡^	144 ± 23^☆Δ†^	714 ± 41^☆Δ^	1428 ± 51^☆Δ‡^
Allo 8W	876 ± 85^*Δ^	894 ± 43^*☆Δ^	882 ± 31^*☆Δ‡^	64 ± 11^*☆Δ†^	1095 ± 81^*☆Δ^	1917 ± 37^*Δ‡^
Allo 12W	294 ± 28^*☆Δ^	426 ± 27^☆Δ^	442 ± 18^☆Δ‡^	63 ± 5^*☆Δ†^	696 ± 44^☆^	1105 ± 29^*☆Δ‡^
Allo24W	89 ± 11^*☆Δ^	170 ± 10^*☆Δ^	243 ± 33^*☆Δ^	53 ± 9^*☆Δ†^	232 ± 17^*☆Δ^	587 ± 23^*‡^
Auto 4W	1550 ± 94^Δ^	3318 ± 342^Δ^	1129 ± 22^Δ†‡^	1599 ± 39^Δ†^	1668 ± 19^Δ^	2156 ± 60^Δ‡^
Auto 8W	1115 ± 84^*^	1609 ± 57^*Δ^	1767 ± 44^*Δ^	1175 ± 97^*†^	1771 ± 27^*Δ^	2081 ± 81^Δ‡^
Auto 12W	605 ± 28^*Δ^	980 ± 67^*^	1547 ± 49^*Δ†^	420 ± 20^*Δ†^	1499 ± 27^*Δ^	1866 ± 30^*Δ‡^
Auto 24W	501 ± 30^*Δ^	775 ± 36^*Δ^	444 ± 9^*Δ†^	309 ± 22^*Δ†^	419 ± 12^*Δ^	553 ± 18^*‡^
−80 °C group	884 ± 52^Δ^	985 ± 22^Δ^	492 ± 36^Δ†^	993 ± 35^Δ^	484 ± 29^Δ^	491 ± 28^Δ^
Control group	1077 ± 41	1160 ± 65	616 ± 20^†^	1164 ± 61	623 ± 17	614 ± 24

*Significantly different from the reconstruction at 4 weeks in the allograft (Allo) and autograft (Auto) groups.

^☆^Significantly different from the autograft group at the same time point in the allograft group.

^Δ^Significantly different from the control group.

^†^Significantly different from the patellar tendon close to the patella at the same time point in the same group.

^‡^Significantly different from the dorsal patellar tendon at the same time point in the same group.

(*p, ^☆^*p*, ^Δ^*p*, ^†^*p*, or ^‡^*p* < 0.05 for all panels, Student’s *t*-test or Mann-Whitney *U* test).

**Table 2:** Comparison of the biomechanical properties the allograft, autograft and control groups (mean ± standard error).

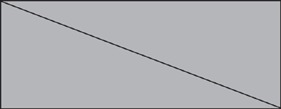	Maximum Load to Failure (N)	Cross-Sectional Area (mm^2^)	Stiffness at Failure (N/mm)	Maximum Stress (N/mm^2^)	Elastic Modulus (Mpa)
Junction of quadriceps tendon-soft tissues					
Allo 4W (n = 3)	50 ± 14.7^Δ^	88.4 ± 8.6	5.2 ± 1.3^Δ^	0.5 ± 0.1^Δ^	1.5 ± 0.2^Δ^
Allo 8W (n = 5)	160.5 ± 16.3^*☆Δ^	65.5 ± 11.7^☆^	21.3 ± 3.8^*☆Δ^	2.9 ± 0.7^*☆^	5.5 ± 1.9*
Allo 12W (n = 3–4)	281.3 ± 7.5^*Δ^	38.6 ± 6.8^*^	46.2 ± 3^*^	7.8 ± 1.3^*^	10.6 ± 0.7^*^
Allo 24W (n = 5)	298 ± 28.7^*^	38.9 ± 4^*Δ^	48.1 ± 3.7^*^	7.8 ± 0.7^*^	8.3 ± 0.7^*^
Auto 4W (n = 3)	65 ± 22.6^Δ^	64.6 ± 12.3	8.3 ± 2.7^Δ^	1.1 ± 0.3^Δ^	2.5 ± 1.1^Δ^
Auto 8W (n = 4)	241.3 ± 24.4^*Δ^	39.2 ± 3.8^Δ^	39.5 ± 5.8^*^	6.5 ± 1.2^*^	6.7 ± 1.5^*^
Auto 12W (n = 3–4)	290.6 ± 26.6^*Δ^	40.8 ± 4.4^∆^	46.7 ± 6.9^*^	6 ± 0.4^*^	8.2 ± 2.4^*^
Auto 24W (n = 3–5)	374 ± 44.4^*^	44.3 ± 3.1^Δ^	47.3 ± 3^*^	7.4 ± 0.7^*^	7 ± 1^*^
Control (n = 19–23)	340.1 ± 11.3	75.8 ± 6.4	42.5 ± 2.9	5.4 ± 0.6	8.2 ± 0.9
Patellar tendon					
Allo 4W (n = 4–5)	68.5 ± 13.8^☆Δ^	26.9 ± 4.4^Δ^	13.4 ± 2.8^☆Δ^	2.6 ± 0.5^☆Δ^	9.3 ± 1.6^☆Δ^
Allo 8W (n = 4)	144.4 ± 13.5^*☆Δ^	22.9 ± 3.1^Δ^	30.2 ± 1.2^*☆Δ^	6.4 ± 0.3^*☆Δ^	19.8 ± 3.7^*Δ^
Allo 12W (n = 4)	221.9 ± 21.1^*☆Δ^	18.9 ± 1.9^Δ^	51.2 ± 3.8^*☆Δ^	11.9 ± 1.1^*Δ^	32.5 ± 3.9^*Δ^
Allo 24W (n = 5)	254 ± 35^*☆Δ^	17.7 ± 0.9^*Δ^	61.2 ± 9.3^*☆Δ^	14.8 ± 2.5^*☆Δ^	49 ± 9.7^*☆Δ^
Auto 4W (n = 4)	129.4 ± 14.2^Δ^	21.3 ± 1.9^Δ^	28.4 ± 3.8^Δ^	6.3 ± 1^Δ^	25.2 ± 10.4^Δ^
Auto 8W (n = 5)	217 ± 17.5^*Δ^	19.5 ± 1.1^Δ^	49.7 ± 5.1^*Δ^	11.4 ± 1.5^*Δ^	28.8 ± 5.3^Δ^
Auto 12W (n = 3)	300 ± 19.8^*Δ^	19.3 ± 2.6^Δ^	68.8 ± 2.7^*Δ^	15.9 ± 1.6^*Δ^	56.7 ± 10.7^Δ^
Auto 24W (n = 5)	345 ± 15.6^*^	15.4 ± 0.7^*Δ^	88 ± 2.5^*Δ^	22.5 ± 0.6^*Δ^	76.7 ± 4^*Δ^
Control (n = 39)	373.2 ± 8.9	13.5 ± 0.3	102.4 ± 2.8	28.3 ± 0.9	118.7 ± 5.8

^*^Significantly different from the reconstruction at 4 weeks in the allograft (Allo) and autograft (Auto) groups.

^☆^Significantly different from the autograft group at the same time point in the allograft group.

^Δ^Significantly different from the control group.

(*p, ^☆^*p*, or ^Δ^*p* < 0.05 for all panels, Student’s *t*-test or Mann-Whitney *U* test).
