# Supplementary material for: Fresh-frozen Complete Extensor Mechanism Allograft versus Autograft Reconstruction in Rabbits
Source: Sci Rep. 2016 Feb 25;6:22106. doi: 10.1038/srep22106 (PMC4766497; doi:10.1038/srep22106)
Supplement: Supplementary Information [file srep22106-s1.pdf]

Supplementary Information

Fresh-frozen Complete Extensor Mechanism Allograft versus Autograft Reconstruction in Rabbits

Guanyin Chen, Hongtao Zhang, Qiong Ma, Jian Zhao, Yinglong Zhang, Qingyu Fan, Baoan Ma*.

Department of Orthopedics, Tangdu Hospital, Fourth Military Medical University, 569 Xinsi Road, Xi'an 710038, China.

* Corresponding author: Tel. /Fax: +86 29 84777433; E-mail address: gukemba@fmmu.edu.cn.

Video S1. Video clip of rabbit activities at 4 weeks postoperatively in the allograft (allo) group. Rabbit can jump well and maintain daily activities.

Video S2. Video clip of rabbit activities at 8 weeks postoperatively in the allograft (allo) group. Rabbit can jump well and maintain daily activities.

Video S3. Video clip of rabbit activities at 12 weeks postoperatively in the allograft (allo) group. Rabbit can jump well and maintain daily activities.

Video S4. Video clip of rabbit activities at 24 weeks postoperatively in the allograft (allo) group. Rabbit can jump well and maintain daily activities.

Video S5. Video clip of rabbit activities at 4 weeks postoperatively in the autograft (auto) group. Rabbit can jump well and maintain daily activities.

Video S6. Video clip of rabbit activities at 8 weeks postoperatively in the autograft (auto) group. Rabbit can jump well and maintain daily activities.

Video S7. Video clip of rabbit activities at 12 weeks postoperatively in the autograft (auto) group. Rabbit can jump well and maintain daily activities.

Video S8. Video clip of rabbit activities at 24 weeks postoperatively in the autograft (auto) group. Rabbit can jump well and maintain daily activities.
